# Periodontal parameters and BANA test in patients with chronic renal failure undergoing hemodialysis

**DOI:** 10.1590/S1678-77572010000300018

**Published:** 2010

**Authors:** Sérgio Aparecido TORRES, Odila Pereira da Silva ROSA, Mitsue Fujimaki HAYACIBARA, Maria do Carmo Machado GUIMARÃES, Roberto M. HAYACIBARA, Walter Antônio BRETZ

**Affiliations:** 1 DDS, MSc, PhD, Assistant Professor, Department of Biological Science, Bauru School of Dentistry, University of São Paulo, Bauru, SP, Brazil.; 2 DDS, MCs, PhD, Associate Professor, Department of Biological Science, Bauru School of Dentistryl, University of São Paulo, Bauru, SP, Brazil.; 3 DDS, MCs, PhD, Professor, State University of Maringa, Maringá, PR, Brazil.; 4 DDS, MCs, PhD, Adjunct Professor, Periodontics Division, University of Brasilia, Brasília, DF, Brazil.; 5 DDS, MCs, PhD, Professor, State University of Maringa, Maringá, PR, Brazil.; 5 DDS, MCs, PHD, Associate Professor, College of Dentistry, New York University, NY, United States.

**Keywords:** Chronic renal insufficiency, Renal dialysis, Periodontal diseases

## Abstract

**Objectives:**

The aim of this study was to analyze the periodontal parameters of patients with chronic renal failure.

**Material and Methods:**

The periodontal status of 16 Brazilian patients aged 29 to 53 (41.7±7.2) years with chronic renal failure (CRF) and another matched group of 14 healthy controls with periodontitis was assessed clinically and microbiologically. Probing pocket depth (PPD), gingival recession (GR), dental plaque index (PLI), gingival index (GI), and dental calculus index (CI) were the clinical parameters recorded for the entire dentition (at least 19 teeth), while the anaerobic periodontopathogen colonization in four sites with the highest PPD was evaluated using the BANA test (“PerioScan”; Oral B).

**Results:**

The results for the CRF group and control group, respectively were: PPD: 1.77±0.32 and 2.65±0.53; GR: 0.58±0.56 and 0.51±0.36; PLI: 1.64±0.56 and 1.24±0.67; GI: 0.64±0.42 and 0.93±0.50; CI: 1.17±0.54 and 0.87±0.52. Comparison between groups using the "t" test revealed a significantly increased PPD (p<0.001) in the control group. Comparison of the other clincial parameters by the Mann-Whitney test showed differences only for PLI, which was significantly higher (p<0.05) in the CRF group. Spearman's test applied to each group showed a positive correlation among all clinical parameters, except for GR (p<0.05). None of the groups showed any correlation between GR and GI, while a significant negative correlation between GR and PPD was observed for the CRF group. The percentage of BANA-positive sites was 35.9% for the CRF group and 35.7% for the control group. The BANA test correlated positively with PPD only in the control group and with GR only in the CRF group.

**Conclusions:**

In spite of a higher PLI and dense anaerobic microbial population even in shallow PPD, patients with CRF exhibited better periodontal conditions than periodontitis patients, which is an evidence of altered response to local irritants.

## INTRODUCTION

Chronic renal failure (CRF) is a progressive, irreversible, bilateral destruction of nephrons resulting from a combination of environmental and genetic causes^11^. Loss of renal function arises with accumulation of metabolic waste products, which in turn change the normal homeostatic mechanisms that control electrolytic balance. Such substances can be removed by the artificial process of dialysis, of which the major goals are to maintain fluid and electrolyte balance and to eliminate waste products from the body ^25^.

Several changes occur in the oral cavity in association with CRF, such as reduction of stimulated salivary flow rate, high salivary urea levels^10,23^ and accelerated rate of calculus accumulation^8^. In spite of these alterations and the certain degree of immunosuppression presented by patients undergoing hemodialysis, chronic renal disease does not appear to be an additional risk for severe periodontitis^17^. This affirmation finds support in the detection of severe periodontitis in a small fraction of the patients undergoing hemodialysis^7^ and in the detection of periodontal parameters, such as the plaque and gingival index and probing depth at levels similar to those in the population in general^17^. Nevertheless, there are data from other studies, whose results are divergent for reporting high prevalence of periodontal disease among patients undergoing hemodialysis^1,19^.

Although it should be considered that systemic diseases can influence the microenvironment of periodontal pockets and potentially affect the composition of subgingival biofilm, analysis of the subgingival microbiota of patients with CRF is scarce in scientific literature^6,11^. In these studies, important periodontopathogens, including *Porphyromonas gingivalis* and *Tanerella forsythensis,* among others, have been detected in patients with renal disease.

Based on the controversies existing in the literature as regards the periodontal conditions of patients undergoing hemodialysis, the aim of this study was to analyze and compare periodontal parameters of patients with CRF to those of systemically healthy controls with periodontitis. In addition, based on the need of evaluating the subgingival microbiota of patients with CRF, this study also investigated whether the presence of BANA-positive pathogens are correlated with the periodontal conditions of these individuals.

## MATERIAL AND METHODS

### Study population

Patients with CRF were selected from the Hemodialysis Division of the Base Hospital in the city of Bauru, SP, Brazil. The inclusion criteria for patients with CRF were to be between 25 and 59 years of age, diagnosed as having CRF, be undergoing hemodialysis for at least 6 months, and present of a minimum of 15 teeth. The exclusion criteria: having fewer than 15 teeth, being younger than 15 or older than 59 years of age, and having received any periodontal treatment or antibiotic therapy in the last six months. Systemically healthy patients with chronic localized or generalized periodontitis selected from the Periodontics Clinic of Bauru Dental School, University of São Paulo, Brazil, served as the control group.

Those who agreed to participate signed an informed consent form approved by the University Institutional Review Board.

### Clinical Examination

Subjects from both groups were examined in a research setting at Bauru School of Dentistry. The clinical parameters were evaluated in all teeth by a single examiner. To evaluate bacterial plaque accumulation and the gingival conditions, the plaque index (PLI) and gingival index (GI) were recorded according to Löe^15^ (1967). To evaluate calculus accumulation, the calculus index (CI) proposed by Anerud A, Löe, Boysen^2^ (1991) was applied. The presence of gingival recession (GR) and pocket probing depth (PPD)^21^ were verified with a “Michigan 0” type millimeter periodontal probe with Williams markings.

In order to obtain reproducibility of the manual PPD measurements, replicated measurements were made in all patients, within a maximum interval of seven days. The mean between the first and second measurements recorded in each site was calculated and taken as the value.

### BANA Test

BANA test (PerioScan; Oral B, Belmont, CA, USA) is a chair-side *in vitro* test which, in subgingival dental plaque samples, can detect the presence of one or more anaerobic bacteria associated with periodontal disease, especially *Treponema denticola, P. gingivalis* and *T. forsythensis*^16^. Following isolation of the area and removal of supragingival plaque with a cotton swab, subgingival plaque samples were collected from 4 sites presenting the deepest PPD using a sterile periodontal curette. Afterwards, plaque samples were placed on BANA (N-benzoylDL-arginine-beta-naphthylamide)-impregnated cellulose paper, processed and read according to the manufacturer’s instructions. The results of this colorimetric test were given by scores: 0- no staining; 1- weak positive (small traces of light blue color), and 2- positive (dark blue color), meaning heavy colonization.

### Statistical analysis

Comparisons between groups were performed according to unpaired “t” test for PPD and Mann-Whitney test for GR, PLI, GI and CI. Spearman’s correlation test was performed to analyze correlations among the different clinical periodontal parameters and among the clinical parameters and a positive BANA test, in the CRF and control groups. The analysis was based on the sites average means. A statistical level of significance was set at p<0.05. The software used for statistical analysis SigmaStat for Windows, version 3.11 (Systat Software, Inc., Chicago, IL, USA).

## RESULTS

Sixteen patients with CRF aged 29 to 53 years (41.7±7.2y) undergoing to hemodialysis for 10 to 88 months (29±22m) and presenting 19 to 29 teeth (23±4t) were selected. The control group was composed of 14 systemically healthy subjects with adult periodontitis, who had 22 to 32 teeth (26±3t), and similar age range and gender distribution.

The characteristics of patients and controls as regards age, gender, number of teeth present and hemodialysis treatment time are shown in Table 1. Mean and standard deviation values for clinical parameters of pocket probing depth, gingival recession, plaque, gingival and calculus indices for both groups are shown in Table 2. The comparison between groups revealed statistically significant differences only for PPD, which was higher in the control group (p<0.001) and for PLI, which was higher in the CRF group (p< 0.05).

**Table 1:** Characterization of patients with chronic renal failure (CRF) and controls

	**CRF group**	**Control group**
		
Age (years)	41.7±7.2	41.4±7.6
Males (%) / Females (%)	75 / 25	71.42 / 28.57
Number of missing teeth	23.3±3.8	25.9±2.8
Duration of hemodialysis	29.1±22.4	______

**Table 2:** Mean ± standard deviation of clinical parameters in patients with chronic renal failure (CRF) and controls

	**CRF**	**Control**	**P value**
			
PPD	1.77±0.32	2.65±0.53	<0.001
GR	0.58±0.56	0.51±0.36	0.755
Pl	1.64±0.56	1.24±0.67	0.036
GI	0.64±0.42	0.93±0.50	0.101
CI	1.17±0.54	0.87±0.52	0.158

With only one exception, Spearman’s correlation test revealed significant correlations among all clinical parameters for both groups. No correlation was observed between GR and GI. A negative correlation between GR and PPD was observed in some sites in the CRF group (Table 3).

**Table 3:** Results of the study of correlations among clinical parameters from 6 sites of the entire dentition in CRF and control groups by Spearman test (p<0.05)

**Probing pocket depth (PPD)**	**Gingival index (GI)**	**Plaque index (PLI)**	**Calculus index (CI)**
			
PPD x PLI *	GI x PLI *	PLI x CI *	CI X GR *
PPD x GI *	GI x CI *	PLI x GR *	
PPD x CI *	GI x GRNS		
PPD x GR **			

CRF= chronic renal failure.

* positive correlation in both groups.

** negative correlation in some areas of the CRF group.

NS= Not significant in both groups.

Table 4 shows BANA test scores observed in the two groups. Positive scores were observed in 23 of 64 sites (35.9%) in the CRF group and in 20 of 56 sites (35.7%) in the control group. The percentage of at least one positive BANA test was 68.8% (11/16 patients) in the CRF group, and 50% in the control group (7/14 patients). The distribution of positive sites relative to their PPD is shown in Table 5. Whereas 46 sites (71.87%) in the CRF group presented PPD<5, 46 sites (82.1%) in the control group presented PPD≥5. Moreover, while BANA positivity in the CRF group was concentrated in PPD between 3 and 5 mm, in the control group this positivity was found in PPD ≥ 5mm.

**Table 4:** BANA test scores in chronic renal failure (CRF) and control groups

**BANA Score**	**CRF **		**Control **	
	**Number**	**sites%**	**Number**	**sites%**
				
0	9	14	13	23
1	32	50	23	41
2	23	36	20	36
Total	64	100	56	100

**Table 5:** Distribution of positive BANA test in different pocket probing depth (PPD) in chronic renal failure (CRF) and control groups

**PPD**	**Control **				**CRF **			
	**Sites**		**BANA +**		**Sites**		**BANA +**	
	**N**	**%**	**N**	**%**	**N**	**%**	**N**	**%**
								
2	___	___	___	___	2	3.1	___	___
3	___	___	___	___	35	54.7	14	61
4	10	17.9	___	___	9	14.1	2	8.7
5	20	35.7	5	25	15	23.4	5	21.6
>5	26	46.4	15	75	3	4.7	2	8.7
Total	56	100%	20	100	64	100%	23	100%

When the correlation between BANA positivity and the clinical parameters PPD and GR was evaluated in each group, a significant correlation was observed between BANA and PPD in the control group, and between BANA and GR in the CRF group. The CRF group presented negative correlation between PPD and GR, whereas the control group presented positive correlation between these clinical parameters.

## DISCUSSION

In this study, patients with CRF exhibited increased PLI and similar CI compared to the control group, composed of patients with periodontal disease. Several studies have reported increased plaque and calculus accumulation in patients with CRF undergoing hemodialysis^1,5,7,17,19^. Al-Wahadni and Al-Omari^1^ (2003) reported that individuals receiving hemodialysis treatment may ignore oral hygiene and other potential problems due to spending a great deal of time at the dialysis center. Therefore, if individuals undergoing dialysis have more difficulty in taking adequate care of oral hygiene, the duration of dialysis could have effects on the periodontal conditions of susceptible individuals. Bayraktar, et al.^3^ (2007) detected high positive correlation between the time of dialysis and GI and PPD. Similarly, Duran and erdemir^7^ (2004) observed that the degree of periodontal destruction increased with the increase of dialysis duration. The authors detected correlation between CPITN and time of duration of dialysis (r=0.240, p<0.01).

The large amount of calculus in patients with CRF could be explained by the high urea content in saliva, which increases the pH after it is metabolized into ammonia; and by the high calcium and phosphate supplement frequently used as part of the dietary control of these patients^13^.

In spite of increased plaque accumulation, GI values in the CRF group were similar to those of the periodontitis group. Rahman, et al.^24^ (1992) recorded lower gingivitis and periodontitis scores in groups of patients undergoing hemodialysis, especially after transplantation, when compared to healthy groups, despite the increased plaque accumulation. It has been suggested that the uremic state may suppress inflammatory reactions in the tissues, which would result in infrequent detection of gingival inflammation in these patients with CRF compared to healthy controls^14^. However, Bots, et al.^5^ (2006) reported a strong correlation between the number of teeth with bleeding and the number of teeth covered with dental plaque and calculus in CRF patients.

The increased prevalence of calculus and plaque in patients with CRF was not reflected in the pocket probing depth. Patients with CRF presented less periodontal breakdown than chronic periodontitis patients. In an epidemiological study about risk indicators for bone loss in periodontal disease, Grossi, et al.^11^ (1995) found a negative correlation with the history of renal disease. According to them, the plausible biological explanation would be that the elevated serum phosphate levels in the disease could contribute to the higher osteoblastic activity.

Another aspect to take into account would be the lower resistance of patients with CRF to infectious diseases due to impaired humoral and cellular immunity and phagocyte functions^20^. Locally, gingival specimens of patients with CRF have been shown to present smaller counts of inflammatory cells than those of healthy subjects^28^. The higher osteoblastic activity mentioned by Grossi, et al.^11^ (1995) may, at least in part, compensate for the deficiency in this important periodontal line of defense.

The two groups showed identical behavior when the correlations between clinical parameters were studied. The role of oral hygiene in periodontal disease can be observed in the direct correlation between PI, CI and PPD in the two groups. Gingival recession presented no correlation with GI in both groups; it presented no correlation with PPD in the control group, and presented negative correlation with PPD in some sites in the CRF group. The multifactorial etiology of GR and its occurrence both in patients with good hygiene and in those with inadequate plaque control^13^ makes it difficult to analyze its correlation with the other clinical parameters of this study. This implicates observation of the etiological factors of recession not evaluated in the study.

The CRF and control groups presented similar positivity (~36%) to the BANA test. Microorganisms related to positivity of this test *(T. forsythensis, Treponema denticola* and *P. gingivalis)* have been associated with high levels of periodontal disease^4^. Moreover, Grossi, et al.^11^ (1995) found an association of *Porphyromonas gingivalis* and *T. forsythensis* with bone and attachment loss, reinforcing their role as indicators of risk for periodontal disease. These pathogens were also described by Socransky, et al.^26^ (1998) as part of the red complex in chronic periodontitis and are related to increased PPD and presence of bleeding on probing.

Notwithstanding similar BANA positive results, it is required to analyze this before both groups PPDs. Whereas 71.9% of the periodontal sites of patients with CRF showed PPD between 2 and 4, only 17.9% of the periodontitis patients had sites with PPD equal to 4. The periodontitis group exhibited increasing BANA positivity with increasing PPD, showing positive correlation (p<0.05) between them; however, patients with CRF presented BANA positivity in every PPD, starting with PPD 3, i.e. 14/35 sites in PPD 3 (40%); 2/9 sites in PPD 4 (22%); 5/15 in PPD 5 (33%) and 2/3 sites in PPD > 5 (66.7%), without correlation between BANA positivity and PPD. The anaerobic environment of deeper pockets in periodontitis contributes to the increasing growth of anaerobic periodontopathogens, while there must be other factors that foster these microorganisms in shallow pockets in CRF. One of these, for example, could be that the subgingival pH is more alkaline in patients with CRF, due to the high systemic urea levels that would be favorable to *P. gingivalis*: laboratory tests with mixed cultures of black anaerobes have demonstrated that an increase in pH from 7.0 to 7.5 may lead to this microorganism increasing in number from being less than 1% of the microbial community to attaining predominance in the culture^19^. The presence of heavy colonization by BANA positive pathogens in all pocket depths as well as the correlation between BANA positivity and GR in patients with CRF, may therefore, indicate that local conditions may somehow affect the microorganisms, either numerically or in virulence.

Moreover, the strong presence of BANA-positive pathogens in the patients with CRF, both in shallow and deeper pockets, points out the importance of discussing the real role of these bacteria in the onset and progression of periodontal disease.

In addition to having established the association between BANA-positive pathogens and greater clinical attachment loss^12^ , longitudinal studies have supported the importance of *T. forsythensis* and P. gingivalis in the onset of chronic periodontitis and in the progression of advanced peridontitis^9^. Nevertheless, the cause-effect relationship between microbiota and periodontitis has not yet been clearly determined. Van Dyke^29^ (2007) suggested that *P. gingivalis* is present in large numbers in periodontitis by colonizing deep pockets. Before the increase in number of the pathogen, a greater inflammatory response would cause tissue breakdown, which would result in increase in periodontal pocket depth and consequent increase in the counts of this microorganism in this environment. Susceptibility to periodontal disease involves factors inherent to the individual, including genetic factors and greater inflammatory response, as well as environmental factors. In individuals susceptible to periodontal disease, chronic exposure to bacterial plaque would result in greater susceptibility^22^. In this context, if on the one hand the detection of *P. gingivalis* in shallow pockets could be related to local factors of the oral environment of these patients, such as the influence of pH on *P. gingivalis* colonization^18^ , while deep pockets would favor the increase in number of these pathogens.

Considering the higher risk of infection in patients with CRF, the observations here reported of massive presence of dental plaque, calculus and microorganisms such as *P. gingivalis* and *T. forsythensis*, even in shallow pockets, associated with several systemic conditions^27^ , must be shared mainly because the possibility of kidney transplant increases the risk of infection in these patients.

## CONCLUSIONS

The following conclusions may be drawn: 1. In spite of having greater bacterial plaque accumulation and heavy calculus accumulation, the patients with CRF presented better periodontal conditions than the control patients with chronic periodontitis. Nevertheless, this finding does not eliminate the need of adequate periodontal treatment and oral hygiene instructions, especially with the aim of minimizing the influence of the periodontopathogenic microbiota in individuals susceptible to periodontitis; 2. The presence of dense populations of BANA-positive pathogens in any PPD in patients with CRF indicates an abnormal local response to irritants; 3. Patients with CRF should be kept on a program of strict oral hygiene control, bearing in mind the risk of infection in future transplants.
